# Solitary subependymal giant cell astrocytoma lacking *TSC1* */2* mutations and TTF‐1 expression: A potential diagnostic pitfall

**DOI:** 10.1111/neup.13013

**Published:** 2024-11-04

**Authors:** Davide Mulone, Andrea Mafficini, Evelina Miele, Francesco Sala, Valeria Barresi

**Affiliations:** ^1^ Department of Diagnostics and Public Health University of Verona Verona Italy; ^2^ Dipartimento di Ingegneria per la Medicina di Innovazione University of Verona Verona Italy; ^3^ ARC‐Net Research Center, University of Verona Verona Italy; ^4^ Oncohematology Research Area, Genetics and Epigenetics of tumors IRCCS Bambino Gesù Children's Hospital Rome Italy; ^5^ Dipartimento di Neuroscienze, Biomedicina e Movimento University of Verona Verona Italy

**Keywords:** DNA methylation, subependymal giant cell astrocytoma, *TSC1/TSC2* mutations, TTF‐1, tuberous sclerosis

## Abstract

Subependymal giant cell astrocytoma (SEGA) is a rare, low‐grade glioma typically associated with tuberous sclerosis (TS) and mutations in the TSC1 or TSC2 genes. It is characterized by an intraventricular location, an expansive growth pattern, and the expression of glial and neural markers. TTF‐1 expression is considered a sensitive marker of SEGA, likely reflecting its origin from progenitor cells in the caudothalamic groove. We report a case of SEGA with unusual immunohistochemical and molecular features in a 20‐year‐old man with no signs or family history of TS. The tumor was located in the anterior horn of the right ventricle and obstructed the foramen of Monro. Histologically, it exhibited an expansive growth pattern and was composed of cells with ovoid nuclei and abundant eosinophilic cytoplasm. Immunohistochemically, the tumor cells were positive for GFAP and S‐100 protein, weakly positive for SOX2, focally positive for synaptophysin, and negative for TTF‐1, neurofilament protein, NeuN, EMA, chromogranin, and BCOR. Scattered OLIG2‐positive neoplastic cells were also observed. Molecular analysis revealed no pathogenic mutations or copy number variations in the analyzed 174 genes, including *TSC1/2*, except for a variant of unknown significance in *BAP1*. The histopathological features and immunohistochemical profile suggested SEGA, despite the absence of TTF‐1 expression and *TSC1/2* mutations. The diagnosis was confirmed by DNA methylation profiling, which assigned the tumor to the methylation class “subependymal giant cell astrocytoma with *TSC1/TSC2* alterations” with a calibrated score of 0.95. This case highlights the potential diagnostic pitfall of SEGA lacking TTF‐1 expression and emphasizes the importance of considering this entity in the differential diagnosis of intraventricular tumors, even in the absence of TS and characteristic molecular alterations. The existence of TTF‐1 negative SEGAs reveals that these tumors might also derive from TTF‐1 negative cells in the subpendymal region.

## INTRODUCTION

Subependymal giant cell astrocytoma (SEGA) is a circumscribed glioma classified as grade 1 according to the World Health Organization (WHO) classification of tumors of the central nervous system (CNS).^1^ Typically, it occurs in individuals in the first two decades of life,^2^ although rare cases have been prenatally diagnosed.^3^ This tumor primarily develops in the cerebral lateral ventricles, where it is supposed to originate from the subependymal tissue in the caudothalamic groove adjacent to the foramen of Monro.^4^

Tuberous sclerosis (TS) is a rare autosomal dominant disorder characterized by pathogenic mutations in TSC1 or TSC2 genes, leading to the formation of hamartomas and benign tumors in the CNS or outside it (e.g., fibromas, angiofibromas, angiomyolipomas, and cardiac rhabdomyomas).^5^ SEGA is the most frequent CNS tumor in patients with TS, and its incidence is reported to be 5–15% in individuals with this syndrome.^5^ Therefore, the presence of this tumor is a major diagnostic criterion for TS.^5^ Almost all SEGAs harbor mutations or deletions in *TSC1* or *TSC2*,^6^ which are tumor suppressor genes that encode hamartin and tuberin, respectively. These two proteins form a heterodimer that suppresses mTOR signaling.^7^ The inactivation of one of these two genes in SEGA leads to the absence of the corresponding protein and a lack of heterodimer formation, resulting in the activation of the mTOR pathway and uncontrolled cell growth.^7^

On magnetic resonance imaging, SEGA commonly presents as a clearly defined, solid mass situated in close proximity to the foramen of Monro and exhibits contrast enhancement.^8^ Histologically, this tumor features an expansive growth pattern and is composed of polygonal cells with abundant cytoplasm, spindle cells, gemistocyte‐like cells, or ganglionic‐like cells that express both glial GFAP and neural (synaptophysin, neurofilament protein, beta‐tubulin III, SOX2, and NeuN) markers.^1^ Some cases exhibit neoplastic cells arranged around vessels, forming a perivascular pseudorosette‐like pattern that might simulate ependymoma. Necrosis, mitoses, and microvascular proliferation can be present, suggesting a malignant glioma; however, these histopathological features are not associated with a worse prognosis in SEGA.^1^, ^9^ Notably, TTF‐1 nuclear immunohistochemical expression is widely regarded as a sensitive marker of SEGA in the differential diagnosis towards histological mimickers,^4^, ^10^, ^11^, ^12^, ^13^ as all SEGAs analyzed in previous studies expressed this transcription factor.^4^, ^10^, ^11^, ^12^, ^13^ TTF‐1 expression in SEGAs is consistent with the possible origin of these tumors from progenitor cells in the medial ganglionic eminence of the caudothalamic groove.^4^ Indeed, this transient anatomical structure of the germinal matrix, located between the caudate nucleus and thalami during embryogenesis^14^ and disappearing by the age of one year, shows TTF‐1 positivity in progenitor cells, which regulates the migration of interneurons to the cortex or striatum.^4^

Herein, we report on a SEGA lacking TTF‐1 immunohistochemical expression and mutations in *TSC1/2*. Acknowledging that SEGAs might lack the expression of this transcription factor and *TSC1/2* mutations is crucial for preventing misdiagnosis.

## CLINICAL SUMMARY

**Fig 1:**
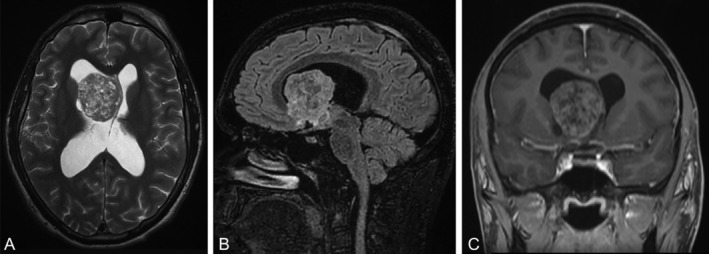
Magnetic resonance imaging findings. (A) An axial T2 image of the brain shows a heterogeneously hyperintense mass in the anterior horn of the right ventricle. (B) A sagittal FLAIR image of the brain shows a heterogeneously hyperintense mass in the anterior horn of the right ventricle. (C) A coronal T1 image of the brain shows a heterogeneously isointense mass with mild contrast enhancement in the anterior horn of the right ventricle.

A 20‐year‐old man underwent computed tomography for craniofacial trauma after a car accident, which unexpectedly revealed a right intraventricular frontomesial lesion. Magnetic resonance imaging confirmed the presence of a mass measuring 46 × 37 × 47 mm in the anterior horn of the right ventricle obstructing the homolateral foramen of Monro, with subsequent supratentorial ventricular dilation. The lesion was heterogeneously hyperintense on T2 and FLAIR sequences and heterogeneously isointense on T1, with mild contrast enhancement (Fig. 1). The patient underwent surgery, with subtotal removal of the lesion, which appeared gray and was implanted on the base of the frontal horn of the right ventricle and in proximity of the thalamus. The residual tumor is currently being followed up, with no evidence of growth after eight months.

The patient had no stigmata of the TS complex. Cutaneous manifestations of TS, including hypomelanotic nodules, facial angiofibroma, or shagreen patches, were absent. Moreover, he had no renal angiomyolipomas, renal cysts, or lung lymphangioleiomyomatosis. The patient's family history was negative for tumors.

## PATHOLOGICAL FINDINGS

**Fig 2:**
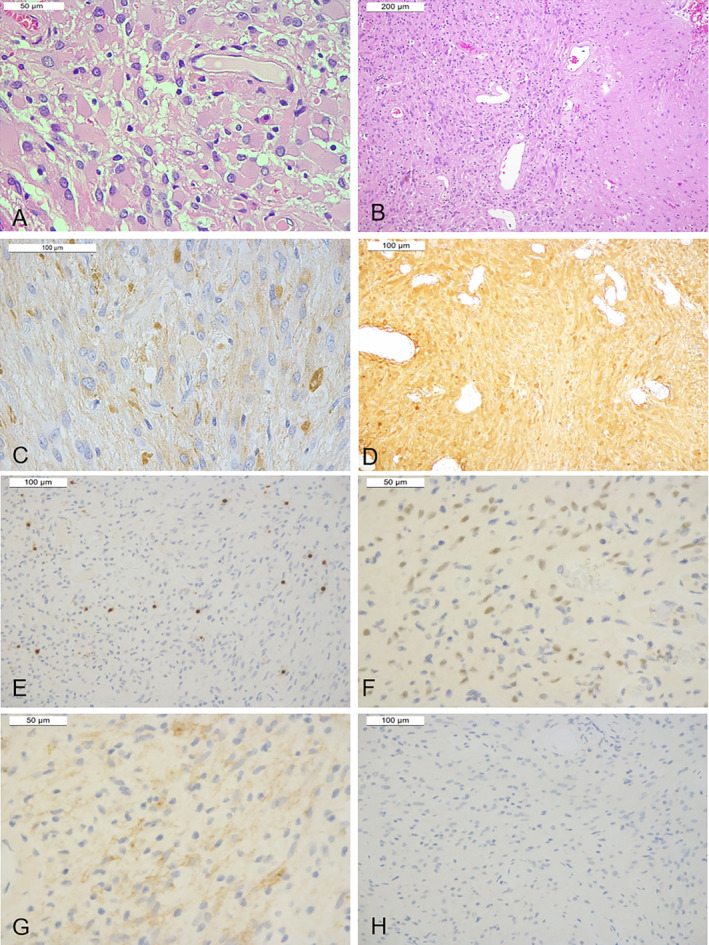
Histological and immunohistochemical features of the tumor on hematoxylin and eosin staining (A, B), GFAP (C), S‐100 protein (D), OLIG2 (E), SOX2 (F), synaptophysin (G), and TTF‐1 (H) immunostainings. (A) The tumor comprises large cells with eosinophilic cytoplasm and nuclei with prominent nucleoli. (B) The tumor has a sharp margin. (C) Tumor cells are positive for GFAP and (D) S‐100 protein. (E) Scattered OLIG2‐positive cells are observed. (F) Weak nuclear immunostaining for SOX 2 and (G) focal immuno‐expression for synaptophysin are present. (H) TTF‐1 is uniformly negative.

Histological examination revealed a moderately cellular tumor with an expansive growth pattern. The tumor was composed of large cells that exhibited ovoid nuclei with dispersed chromatin and prominent nucleoli and abundant eosinophilic cytoplasm. Frequent Rosenthal fibers were also observed (Fig. 2). Mitoses, necrosis, and microvascular proliferation were absent.

Immunohistochemical analysis revealed that the tumor cells exhibited diffuse positivity for GFAP (mouse monoclonal, clone GA5, prediluted; Leica Biosystems, IL, USA) and S‐100 protein (rabbit polyclonal; DAKO Agilent, CA, USA; 1:3000), mild positivity for SOX2 (rabbit polyclonal; SEven Hills Bioreagents, OH, USA; 1:2000), and focal positivity for synaptophysin (mouse monoclonal, clone 27G12; Leica Biosystems, IL, USA; prediluted) (Fig. 2). OLIG2 was positive in scattered neoplastic cells (rabbit monoclonal, clone EPR2673; Abcam, Italy; 1:100). The tumor cells were negative for TTF‐1 (mouse monoclonal, clone SPT24; Leica Biosystems, IL, USA; prediluted), neurofilament protein (mouse monoclonal, clone 2F11; DAKO Agilent, CA, USA; 1:150), NeuN (mouse monoclonal, clone 1B7; Abcam, Italy; 1:500) (Fig. 2), EMA (mouse monoclonal, clone E29; DAKO Agilent, CA, USA; 1:400), chromogranin (mouse monoclonal, clone DAK‐A3; DAKO Agilent, CA, USA; 1:50), CD‐34 (mouse monoclonal, clone QBEND/10; Leica Biosystems, IL, USA; prediluted), and BCOR (mouse monoclonal, clone BSB128; BIO‐SB, CA, USA; 1:20) and retained ATRX immunoexpression (mouse monoclonal, clone X1; Dianova, Germany; 1:200). The Ki‐67 labelling index (mouse monoclonal, clone MN1; Leica Biosystems, IL, USA; prediluted) was 1%.

**Fig 3:**
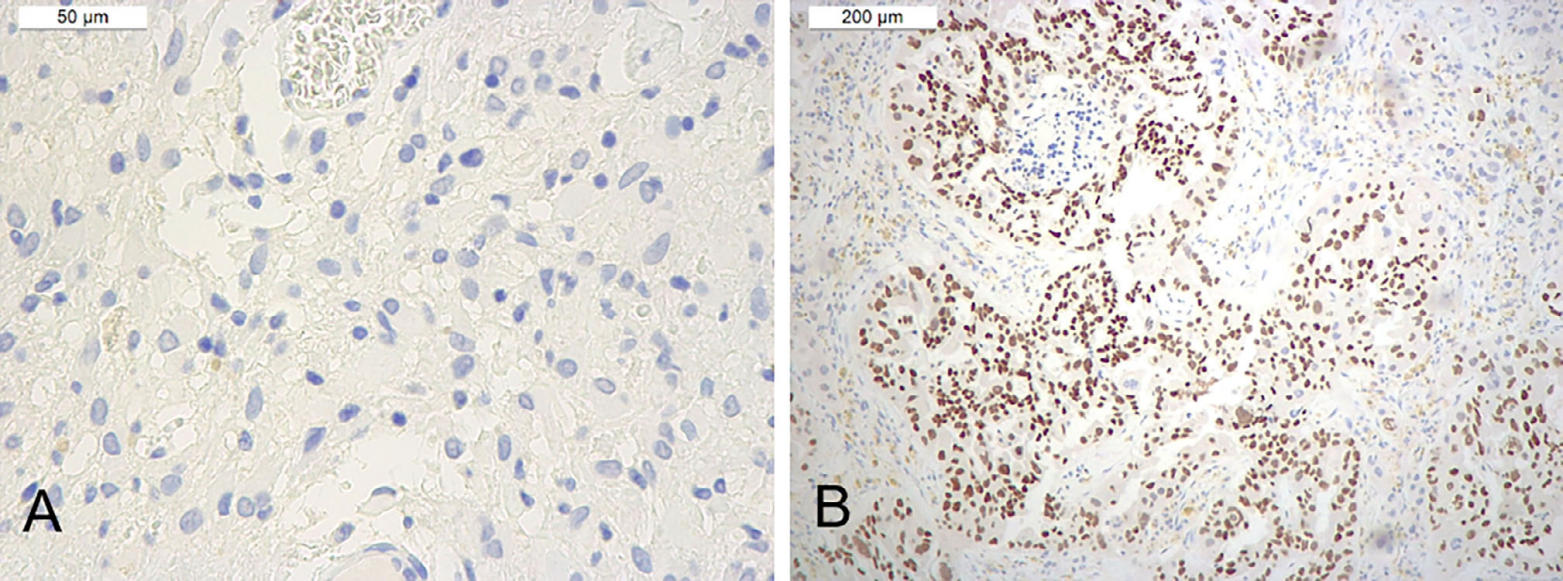
Immunohistochemical features of the tumor on TTF‐1 immunostaining using anti‐TTF1 antibody clone 8G7G3/1. The tumor cells of SEGA are negative for TTF‐1 (A), whereas nuclear positivity for TTF‐1 is present in the neoplastic cells of a brain metastasis of lung adenocarcinoma used as positive control (B).

TTF‐1 immunostaining was repeated using a different clone of the monoclonal antibody against TTF‐1 (mouse monoclonal, clone 8G7G3/1; Cell Marque, Rocklin, CA, USA; 1:200); tumor cells were again negative, whereas nuclear positivity was present in the neoplastic cells of a brain metastasis of lung adenocarcinoma used as positive control **(**Fig. 3 **)**.

## MOLECULAR FINDINGS

Using fluorescence in situ hybridization, no rearrangements of *ZFTA* (break‐apart probe, Empire Genomics, New York, USA), *YAP‐1* (break‐apart probe, Empire Genomics, New York, USA), *MN1* (break‐apart probe, Empire Genomics, New York, USA), and *BRAF* (break‐apart probe, ZytoVision, Bremerhaven, Germany) were observed.

The tumor was additionally analyzed using the SureSelectXT HS CD Glasgow Cancer Core assay (www.agilent.com), which spans 1.85 Mb of the genome and interrogates 174 genes (listed in Fig. S1) for somatic mutations, copy number alterations, and structural rearrangements.^15^ We did not find any pathogenic mutations or copy number variations in the analyzed genes, including *IDH‐1*, *IDH‐2*, *H3F3A*, *HIST1H3B*, *TSC1*, *TSC2*, *BRAF*, or *NF1*, with the exception of the c.783 + 2 T > C mutation in *BAP1* (NM_004656; variant allele frequency: 48%), which was classified as a variant of unknown significance.^16^

**Fig 4:**
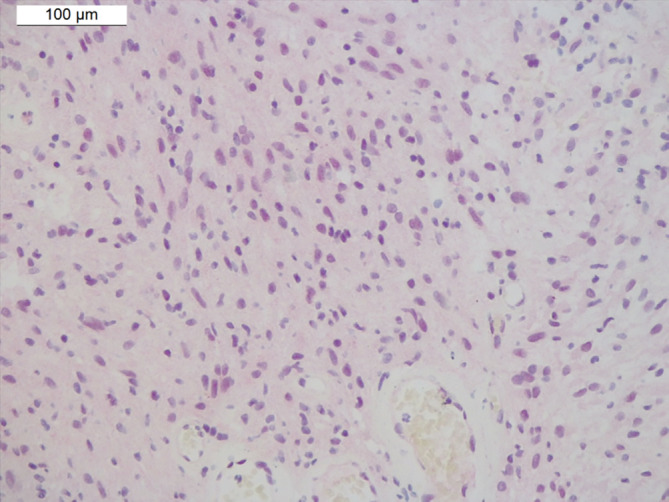
Immunohistochemical features of the tumor on BAP1 immunostaining. Tumor cells show weak nuclear staining for BAP1.

The histopathological features (cell morphology and expansive pattern), location in the lateral ventricle, GFAP positivity, and focal staining for OLIG2 and synaptophysin suggested SEGA. Immunohistochemistry for BAP1 protein (clone C4, dilution 1:100; Santa Cruz Biotechnology, Germany), performed due to the *BAP1* mutation, showed nuclear staining for this protein with a milder intensity than that found in normal cells **(**Fig. 4 **)**.

Owing to the unusually negative immunostaining for TTF‐1 and lack of mutations in *TSC1* and *TSC2*, DNA methylation analysis was performed using the Infinium Methylation EPIC BeadChip (850 k) array (Illumina), as previously described.^17^ Using the methylation classifier v. 12.5 (available at “http://www.molecularneuropathology.org/”), the tumor was assigned to the methylation class “subependymal giant cell astrocytoma with *TSC1/TSC2* alterations,” with a calibrated score of 0.95, and had a flat copy number profile.

## DISCUSSION

In the WHO 2021 classification of CNS tumors, SEGA is listed among circumscribed gliomas.^1^ This tumor mostly arises in the context of TS, and its occurrence is one of the major diagnostic criteria for this syndrome.^18^ Rare SEGAs, designated as solitary SEGA, non‐TS SEGA, or sporadic SEGA, arise in patients who do not have other signs of TS complex^19^ but harbor germline mutations in *TSC1* or *TSC2*, still consistent with TS. More rarely, solitary SEGAs display somatic mutations in *TSC1/2* in the absence of germline mutations, which suggests TS with somatic mosaicism.^19^, ^20^ To the best of our knowledge, only three SEGAs devoid of mutations or deletions in *TSC1* or *TSC2* have been reported,^21^, ^22^, ^23^ and one of these cases had an atypical location in the occipital horn of the lateral right ventricle.^23^

Histologically, SEGA is a circumscribed tumor composed of spindle or plump gemistocyte‐like cells with abundant eosinophilic cytoplasm, a large nucleus, and a prominent nucleolus^1^ and features concurrent immunohistochemical expression of glial and neuronal markers.^1^ Similar histopathological and immunohistochemical features have been described in a subset of astrocytic tumors occurring in patients with NF1, designated as SEGA‐like astrocytomas.^24^ Notably, these tumors harbor mutations in *NF1* and rarely feature *TSC2* mutations.^24^ The differential diagnosis between SEGAs and SEGA‐like astrocytomas relies mainly on clinical features. DNA methylation analysis has proven useful for classifying CNS tumors and for the differential diagnosis of cases with overlapping morphology.^25^ As SEGA has a unique and distinctive DNA methylation profile, DNA methylation analysis can be used to distinguish this tumor from histological mimickers. For instance, in a recent study, DNA methylation profiling classified a SEGA‐like tumor, arising in a 75‐year‐old woman without NF1 or TS, as glioblastoma *IDH* wild‐type with a high calibrated score of 0.99.^26^

Here, we report a case of SEGA lacking *TSC1* and *TSC2* mutations in a patient with no signs or family history of TS. This tumor showed unusual immunohistochemical findings. Indeed, the neuronal markers NeuN, neurofilament protein, and chromogranin were negative, and the tumor cells displayed mild positivity for SOX2 and focal immunoreactivity for synaptophysin. In addition, in contrast to the 68 SEGAs previously analyzed,^4^, ^10^, ^11^, ^12^, ^27^, ^28^ the tumor cells were uniformly negative for TTF‐1, using either clone SPT24 or clone 8G7G3/1 of the antibody against TTF‐1.

In previous studies, TTF‐1 expression in SEGAs was tested using these two different clones of the TTF‐1 antibody. All tested cases were positive for TTF‐1, independent of the clone used.^4^, ^27^ Although clone SPT24 is considered to be less specific, because TTF‐1 expression using this clone was rarely found in diffuse gliomas, ependymomas or choroid plexus carcinomas,^29^ it displays higher sensitivity than clone 8G7G3/1 in detecting TTF‐1 expression in SEGAs. Indeed, in seven SEGAs, TTF‐1 immunostaining was strong and diffuse in clone SPT24 and patchy in clone 8G7G3/1.^27^

In the present case, the diagnoses of ependymoma and *MN1*‐altered astroblastoma were excluded because of the lack of perivascular pseudorosettes and *ZFTA*, *YAP1*, and *MN1* fusions. Although the patient did not harbor any signs or family history of NF1, to exclude a SEGA‐like astrocytoma, we performed DNA methylation profiling, which definitely classified this case as SEGA. This represents the first reported SEGA lacking *TSC1/2* mutations occurring outside the TS complex and was confirmed by a consistent DNA methylation profile.

Owing to TTF‐1 positivity, SEGA was previously thought to originate from progenitor cells of the ganglionic eminence that persisted after birth, which also express TTF‐1.^4^ However, the present TTF‐1 negative SEGA suggests possible alternative derivations of this tumor.

Notably, while next‐generation sequencing of this case did not show any *TSC1/2* mutations, it revealed the c.783 + 2 T > C mutation in *BAP1*. This was previously reported as a germline mutation in two individuals with mesothelioma and cutaneous melanoma, who were suspected to have BAP‐1 tumor predisposition syndrome.^30^ This hereditary condition is characterized by a predisposition to develop different tumor types, including mesothelioma, cutaneous, and uveal melanoma and, among CNS tumors, meningioma.^30^ However, owing to discrepant findings, the significance of the *BAP1* c.783 + 2 T > C mutation remains unknown.^16^

In conclusion, this report presents the first documented SEGA confirmed by DNA methylation analysis that lacks *TSC1* and *TSC2* mutations, in a patient without signs of TS. This suggests that additional molecular alterations might contribute to the development of this tumor. Although TTF‐1 nuclear immunoexpression is generally considered a sensitive marker of SEGA and is listed among the desirable diagnostic criteria for SEGA by WHO,^1^ our study showed that TTF‐1‐negative SEGAs can still occur, potentially originating from cells that do not express TTF‐1. Therefore, the absence of this marker does not necessarily rule out the diagnosis of SEGA. Additionally, this report presents the first case of SEGA with a *BAP1* mutation, although the significance of this finding requires further investigation.

## DISCLOSURES

Funding for this study was provided by the Italian Ministry of University, Progetto PRIN 2022 “Radiological, molecular and histological study of glioblastoma IDH wild‐type characterized by RB1 alteration: Towards the identification of a new variant potentially responsive to immunotherapy.” The funding source had no role in the design, practice, or analysis of this study.

The authors declare no conflicts of interest.

## CONSENT FOR PUBLICATION

The patient signed informed consent for using surgical material and anonymous health information for scientific purposes and publication.

## Supporting information


**Supplementary Figure S1.** List of genes included in the CORE targeted sequencing assay.

## Data Availability

The data that support the findings of this study are available from the corresponding author upon reasonable request.
