# Supplementary figures and images for: Solitary subependymal giant cell astrocytoma lacking TSC1 /2 mutations and TTF‐1 expression: A potential diagnostic pitfall
Source: Neuropathology. 2024 Nov 4;45(2):167–73. doi: 10.1111/neup.13013 (PMC11962586; doi:10.1111/neup.13013)

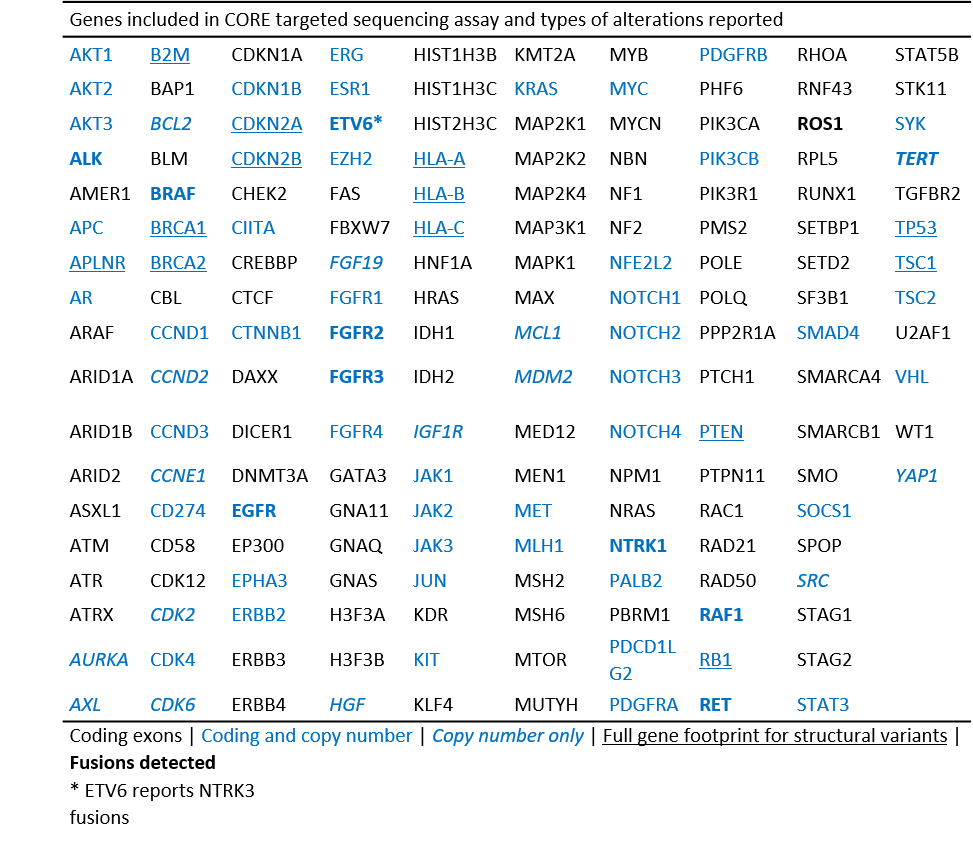

Supplement: Supplementary file 1 — Supplementary Figure S1. List of genes included in the CORE targeted sequencing assay. [file NEUP-45-167-s001.png]
